# Reference Limits for Fetal Biventricular Longitudinal Strain Using Speckle Tracking Echocardiography Across Gestational Age Groups: A Single-Center Study

**DOI:** 10.3390/jcm14155226

**Published:** 2025-07-24

**Authors:** Andreea Cerghit-Paler, Amalia Fagarasan, Dorottya Gabor-Miklosi, Claudiu Mărginean, Mihaela Iancu, Liliana Gozar

**Affiliations:** 1 Doctoral School, George Emil Palade University of Medicine, Pharmacy, Science, and Technology of Târgu-Mureș, 540139 Târgu-Mureș, Romania; palerandreea@yahoo.com (A.C.-P.); annadorka@yahoo.com (D.G.-M.); 2 Department of Pediatrics III, George Emil Palade University of Medicine, Pharmacy, Science, and Technology of Târgu-Mureș, 540139 Târgu-Mureș, Romania; amalia.fagarasan@umfst.ro (A.F.); liliana.gozar@umfst.ro (L.G.); 3 Emergency Institute of Cardiovascular Diseases and Transplantation, 540139 Târgu-Mureș, Romania; 4 Department of Obstetrics and Gynecology, George Emil Palade University of Medicine, Pharmacy, Science, and Technology of Târgu-Mureș, 540139 Târgu-Mureș, Romania; claudiu.marginean@umfst.ro; 5 Department of Medical Informatics and Biostatistics, Iuliu Hațieganu University of Medicine and Pharmacy Cluj-Napoca, 400349 Cluj-Napoca, Romania

**Keywords:** fetal myocardial function, speckle tracking echocardiography, fetal echocardiography, fetal myocardial strain

## Abstract

**Background/Objectives:** The development of normal fetal cardiac function, a dynamic process that has not yet been precisely documented throughout the literature, is difficult to quantify by classic echocardiography. Our aim was to analyze the function of the fetal myocardium through speckle tracking and establish reference values for global and segmental longitudinal strain for both ventricles in fetuses with a gestational age (GA) between 22 and 39 weeks. **Methods:** We conducted a prospective study in which 170 fetuses underwent echocardiographic evaluation and those 150 that were eligible for the study underwent offline speckle tracking analysis. **Results:** A mixed-design ANOVA model with Greenhouse–Geisser correction showed no significant differences in regional strain measurements among GA groups (F [2, 147] = 1.25, *p* = 0.289) but showed significant differences in regional strain measurements among the right ventricle (RV), left ventricle (LV), and interventricular free wall (Greenhouse–Geisser F [1.3, 195.2] = 45.70, *p* < 0.001, GG ε = 0.66, original df = 2, 294). The wall-by-segment interaction term of the model was statistically significant for regional strain (Greenhouse–Geisser F [2.7, 394.2] = 27.00, *p* < 0.001, GG ε = 0.67, original df = 4, 588), while the segment-by-gestational age group term had a tendency toward statistical significance (Greenhouse–Geisser F [3.0, 221.4] = 2.21, *p* = 0.088, GG ε = 0.75, original df = 4, 294). The results of Welch’s ANOVA model showed no significant difference in right-ventricle peak global longitudinal strain (pGLS) between GA groups (F [2.0, 92.2] = 0.52, *p* = 0.5972) and global longitudinal strain measurements (F [2.0, 89.6] = 27.00, *p* = 0.3733). **Conclusions:** The reference values for longitudinal strain, represented by the pGLS for LV, ranged from −20.79 to −8.05 for fetuses with a GA between 22 and 27 weeks, from −20.14 to −8.99 for fetuses with a GA between 28 and 33 weeks, and from −20.19 to −8.88 for fetuses with a GA between 34 and 39 weeks. For RV pGLS, the reference values were between −18.99 and −6.35, also depending on GA. Reference ranges for the large gestational groups studied can help us to recognize subtle changes in fetal cardiac function.

## 1. Introduction

As fetuses are being referred to as patients, alongside the improvement of both the diagnosis and treatment of cardiac pathologies, we are in need for more advanced imaging methods in the evaluation of fetal status [1]. The development of diagnostic and treatment methods during the intrauterine period implies the need for the objective quantification of myocardial function. Using classic echocardiography, the longitudinal and regional evaluation of the myocardium is difficult and subjective [2]. In recent years, several studies and advancements have been made regarding fetal speckle tracking echocardiography, as this provides additional information to that of classic echocardiography in the evaluation of cardiac function [3].

Speckle tracking evaluates the global and segmental deformation of the ventricular myocardium (strain). In the fetal period, multiple factors, such as fetal movement, increased heart rate, low blood pressure, the small size of the fetal heart, the dynamic physiological process of cardiac transition, or the physiological maturation of the myocardium, the presence of some pathologies (poly/oligo-hydramnios, maternal obesity), can produce a distortion in echocardiographic image quality, resulting in measurement errors and thus impacting the final analysis. Therefore, deformation parameters such as myocardial strain provide a quantitative technique for the estimation of global and segmental myocardial function. Currently, peak longitudinal global LV strain is the parameter most often used for the analysis of segmental and global myocardial function in adults. Speckle tracking overcomes the limitations imposed by classical echocardiography (such as dependency on the image acquisition angle), thus offering a special advantage in fetal echocardiography [4].

For the routine use of fetal speckle tracking, but also for the recognition of subtle changes in fetal cardiac function, reference values must be obtained. Due to the dynamic process of normal cardiac function during fetal development, these parameters are difficult to establish precisely. Medjedovic E. et al. conducted an extensive review from which they concluded that the ascertainment of reference values for myocardial strain in the second trimester of pregnancy, by speckle tracking echocardiography, in a cohort of healthy fetuses is essential in the evaluation of myocardial pathologies during pregnancy [5].

The aim of this study was to analyze the function of the fetal myocardium through speckle tracking and establish reference values for global and segmental longitudinal strain in both ventricles in fetuses with a gestational age between 22 and 39 weeks.

## 2. Materials and Methods

**Figure 1:**
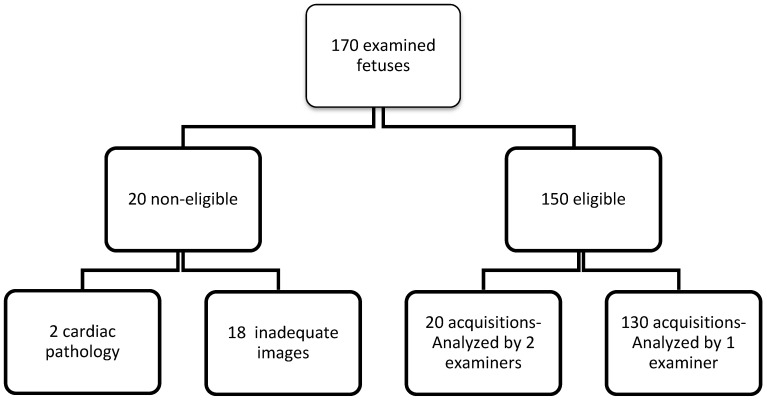
Study flow-chart.

We conducted a prospective, single-center study in the Pediatric Cardiology Department of the Emergency Institute for Cardiovascular Diseases and Heart Transplant in Targu-Mures, Romania, between 27 February 2021 and 27 August 2023, a time frame in which 170 fetuses underwent echocardiographic evaluation, with gestational ages ranging between 22 and 39 weeks. The inclusion criteria for this study were as follows: fetuses with a normal weight for their gestational age and physiological, singleton pregnancies free from both maternal and fetal pathologies. Fetuses with intrauterine growth retardation, cardiac or extracardiac malformations, or rhythm or conduction disorders; twin pregnancies; pregnancies affected by maternal pathologies (diabetes, hypertension); and cases in whom the echocardiographic acquisitions were suboptimal (maternal obesity, difficult fetal position), respectively in whom acquisition of 4-chambers view images with a framerate over 80 was not possible were excluded from the study (Figure 1).

The selection of fetuses was carried out in conformity with the guidelines of The International Society of Ultrasound in Obstetrics and Gynecology (ISUOG) by an obstetrician with expertise in maternal–fetal medicine, who excluded any maternal pathology and made an estimation of fetal growth accordingly [6].

Fetal echocardiograms were performed by a pediatric cardiologist with experience in fetal cardiology using a Philips Epiq 7 ultrasound scanner (Philips, Bothell, WA, USA). A comprehensive echocardiographic evaluation was performed to exclude both structural and functional cardiac pathologies. For each fetus, a minimum of three 2D fetal echocardiographic acquisitions were obtained from an apical 4-chamber view, with a frame rate above 90 Hz. The images were later stored in Digital Imaging and Communications in Medicine (DICOM) format.

### 2.1. Speckle Tracking Analysis

From the stored DICOM images, those of superior quality were selected and analyzed offline, using Philips Qlab 15.0 (material number 453562090801) software with the left- and right-ventricular autostrain function. In the absence of a fetal ECG, the cardiac cycle was manually identified by the operator, based on the mechanical movements of the mitral valve (its complete closure defined end diastole). Following the manual identification of the cardiac cycle, the software calculated the heart rate, which was subsequently compared with the value obtained from the Doppler examination. After establishing the cardiac cycle, the operator selected 3 points from the level of the endocardium in a well-defined order. Firstly, the base of the septum was marked, followed by the lateral edge of the mitral ring, and the third point given was the apex. Based on the 3 points, the software automatically generated the edge of the endocardium, a marking that went on to be checked and corrected manually afterwards. The speckle tracking parameters generated were those of regional strain (basal, medial, and apical IVS; basal, medial, and apical LV; and basal, medial, and apical RV), global biventricular strain (RVAP4pLS, LVAP4pLS), biventricular volumes (end-diastolic volume, EDV; end-systolic volume, ESV), biventricular ejection fraction (EF), and biventricular R time. These parameters were included in the database and analyzed statistically. Although the echocardiographic evaluation was performed by a single examiner, a number of 20 acquisitions were randomly examined offline by a second operator, using the same protocol.

Speckle tracking echocardiography estimates EDV and ESV by tracking myocardial speckles throughout the cardiac cycle to delineate endocardial contours frame by frame. The software uses these traced borders to calculate ventricular areas at end diastole and end systole. It then applies geometric assumptions to convert these area measurements into volumes. Because speckle tracking provides accurate frame-by-frame endocardial tracking independent of the Doppler angle, it enhances reproducibility and objectivity in volume calculations compared to manual tracing alone [7].

To estimate the function of the LV, we proposed the determination of volumetric EF. In practice, this is the main method for quantifying LV systolic function and represents the percentage of the volume ejected in systole (stroke volume, SV) in relation to the EDV. SV is the difference between EDV and ESV. LVEF is calculated as follows: [SV/EDV] × 100 [7]. Considering the structure and geometry of the RV, we did not consider it correct to use this calculation method, the RV function thus being evaluated only by speckle tracking.

Strain is a method of quantifying myocardial deformation in response to the application of a force. An object can increase or decrease its length when a force is applied to it. Strain is measured as the difference between the final and initial length, relative to its initial length. Negative values for myocardial strain describe shortening (systole), and positive values describe lengthening (diastole). The more negative the values, the more efficient the shortening (implicit systole). GLS represents the sum of longitudinal ventricular segmental strains [8]. LVTTPV (ms) and RVTTPV (ms) represent the variability of the times in which each segment reaches maximum elongation. We considered that the high variability of these times suggests an asynchrony between different ventricular segments, an asynchrony that can be reflected in the ventricular function.

### 2.2. Ethics

The entire research protocol was conducted in accordance with the principles stated in the declaration of Helsinki. An informed consent form detailing the methodology of the study was provided to each pregnant woman enrolled in the study to sign. Those women who refused to sign the informed consent form were automatically excluded from the study. The present research was approved by the Ethical Committee of the Emergency Institute of Cardiovascular Disease and Heart Transplantation from Targu Mures and “George Emil Palade” University of Medicine and Pharmacy Targu-Mures 1276/25 February 2021.

### 2.3. Statistical Analysis

The description of anthropometric and echocardiographic characteristics in each of three gestational age groups (group 1, 22–27 weeks; group 2, 28–33 weeks; and group 3, 34–39 weeks) was performed using the arithmetic mean (standard deviation) or median (interquartile range) as a centrality (variability) measure.

Comparisons of regional strain measurements across gestational age groups were conducted using a mixed-design ANOVA model with an interaction term between within-subjects factors (wall and region) and the between-subjects factor (gestational age group), while longitudinal strain measurements were compared using Welch’s ANOVA model across gestational age groups. A significant result with the mixed-design ANOVA model was followed by a post hoc analysis using the Tukey test.

Interobserver agreement was evaluated using intraclass correlation coefficients (ICCs) with 95% confidence intervals and Bland–Altman analysis. Estimated values of ICCs were interpreted as follows: an ICC ≥ 0.90 suggested excellent reliability, 0.75 ≤ ICC < 0.90 indicated good reliability, 0.50 ≤ ICC < 0.75 indicated moderate reliability, and an ICC < 0.50 suggested poor reliability [9].

The reference intervals for regional and longitudinal strain measurements in each gestational age group were calculated by a robust method. The robust method (Horn’s method) was used to estimate reference intervals, as the sample size within each gestational age group was moderate (40 < n < 120). The robust method incorporated outlier detection based on Mark van der Loo’s approach and 90% confidence intervals (90% CIs) for the 2.5th and 97.5th percentiles. The 90% CIs were estimated using the bootstrap percentile method with 5000 resamples, ensuring stable and reliable interval estimation.

We also tested the distributions of time to peak variability (TTPV), defined as the standard deviations of values across gestational age groups, using the Kruskal–Wallis test and their correlation with the ejection fraction (EF) in each gestational age group using Spearman’s correlation coefficient (ρ).

All two-sided statistical tests used a significance level (α) chosen to be 0.05. In the current study, *p*-values < 0.05 denoted a statistically significant result, while 0.05 ≤ *p* < 0.10 was marginally significant.

Statistical analysis was performed in R software version 4.4.0 (R Foundation for Statistical Computing, Vienna, Austria).

## 3. Results

### 3.1. Description of Study Groups

A total of 150 pregnant women were eligible for the current study, and their fetuses were divided into three gestational age groups: 22–27 weeks (group 1, n_1_ = 46), 28–33 weeks (group 2, n_2_ = 55), and 34–39 weeks (group 3, n_3_ = 49). The distributions of the demographic, anthropometric, and clinical characteristics of fetuses across the three gestational age groups are described in Table 1. No significant differences in maternal age (*p* = 0.1389) or femur length (*p* = 0.6270) and biparietal diameter (*p* = 0.8170) were detected between the study groups.

### 3.2. Comparisons of Regional and Global Longitudinal Strain Measurements Across Gestational Age Groups

The results of the mixed-design ANOVA model showed no significant differences in regional strain measurements among gestational age groups (F [2, 147] = 1.25, *p* = 0.289, generalized η^2^ = 0.010). The results of the mixed-design ANOVA model with Greenhouse–Geisser correction highlighted significant differences in regional strain measurements among the RV, LV and interventricular free wall (Greenhouse–Geisser F [1.3, 195.2] = 45.70, *p* < 0.001, GG ε = 0.66, original df = 2, 294, generalized η^2^ = 0.052). The wall-by-segment interaction term of the model was statistically significant for regional strain (Greenhouse–Geisser F [2.7, 394.2] = 27.00, *p* < 0.001, GG ε = 0.67, original df = 4, 588 generalized η^2^ = 0.024), while the segment-by-gestational-age-group term had a marginal statistical significance (Greenhouse–Geisser F [3.0, 221.4] = 2.21, *p* = 0.088, GG ε = 0.75, original df = 4, 294, generalized η^2^ = 0.003). Post hoc pairwise tests based on the Tukey method of the model-estimated means were used to explore these interactions. We found statistically significant differences in the means of regional strain measurements between the basal and middle segments (adjusted-*p* = 0.0427) of the LV free wall, apical, and basal segments (adjusted-*p* < 0.0001) and the medial and basal segments (adjusted-*p* < 0.0001) of the RV free wall.

The results of Welch’s ANOVA model showed no significant difference in right-ventricle peak global longitudinal strain between gestational age groups (F [2.0, 92.2] = 0.52, *p* = 0.5972) and global longitudinal strain measurements (F [2.0, 89.6] = 27.00, *p* = 0.3733).

**Figure 2:**
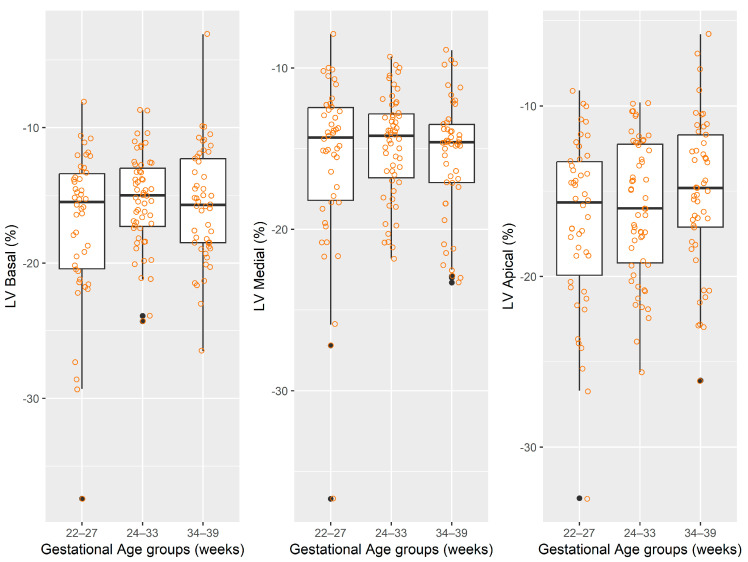
The distribution of values for left-ventricle strain measurements. Note: for each box-and-whisker plot, the box contains the data range from the lower to upper quartile (25th to 75th percentile), while the middle line denotes the median, and the points represent the measured strain values. The black dot points represent outliers automatically detected by the boxplot method.

**Figure 3:**
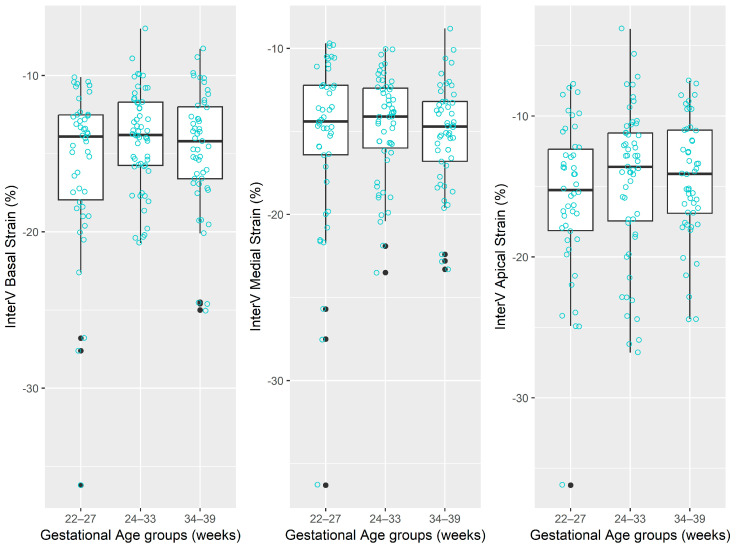
The distribution of values for interventricular septum strain measurements. Note: for each box-and-whisker plot, the box contains the data range from the lower to upper quartile (25th to 75th percentile), while the middle line denotes the median, and the points represent the measured strain values. The black dot points represent outliers automatically detected by the boxplot method.

**Figure 4:**
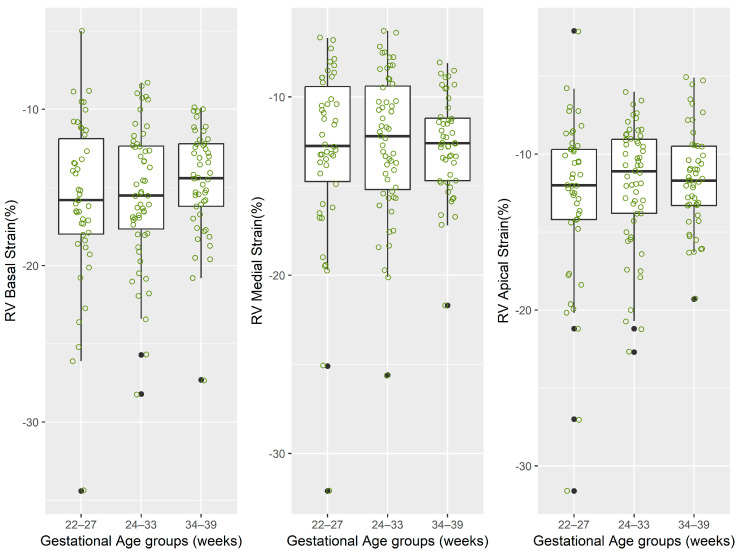
The distribution of values for right-ventricle strain measurements. Note: for each box-and-whisker plot, the box contains the data range from the lower to upper quartile (25th to 75th percentile), while the middle line denotes the median, and the points represent the measured strain values. The black dot points represent outliers automatically detected by the boxplot method.

**Figure 5:**
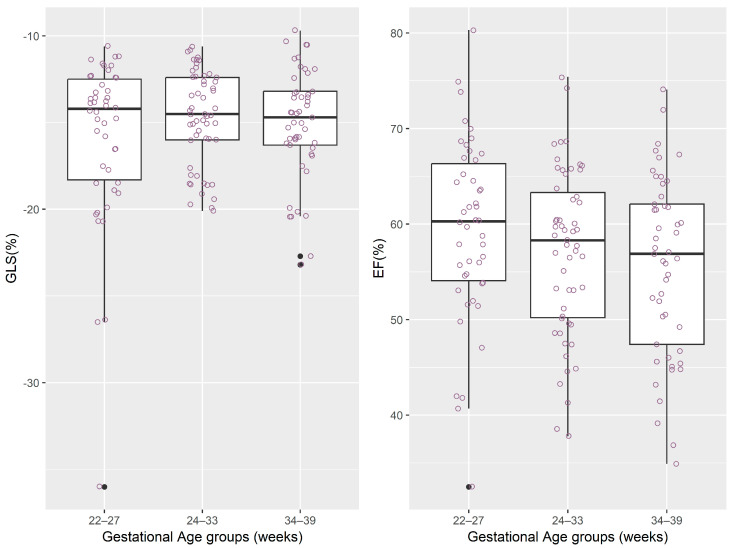
The distribution of values for longitudinal strain measurements and ejection fraction. Note: for each box-and-whisker plot, the box contains the data range from the lower to upper quartile (25th to 75th percentile), while the middle line denotes the median, and the points represent the measured values. The black dot points represent outliers automatically detected by the boxplot method.

The distributions of regional and global longitudinal strain measurements stratified by gestational age groups are depicted graphically in Table 2 and Figure 2, Figure 3, Figure 4 and Figure 5.

### 3.3. Interobserver Reproducibility Analysis

The ICC estimated values were moderate to good for interobserver variability for all echocardiographic parameters. Bland–Altman analysis revealed no significant systematic bias between two observers as the 95% confidence intervals for bias included zero (Table 3).

### 3.4. Reference Intervals for Regional and Global Longitudinal Strain Measurements Across Gestational Age Groups

The reference limits and their 90% confidence intervals estimated using a robust method for regional and global longitudinal strain measurements stratified by gestational age groups are presented in Table 4.

### 3.5. Additional Analysis

#### 3.5.1. Distributions of Time-to-Peak-Variability (TTPV) Values Among Gestational Age Groups

In addition, we found no significant differences in the time to peak variability values of the LV between the gestational age groups studied (median [IQR]: 7.45 [4.43, 21.88] for group 1, 8.20 [3.85, 20.65] for group 2, 13.20 [5.00, 22.10] for group 3, Kruskal–Wallis test, *p* = 0.5245) and the RV free wall (median [IQR]: 12.85 [3.15, 26.05] for group 1, 10.00 [3.15, 23.50] for group 2, 7.70 [3.80, 27.50] group 3, Kruskal–Wallis test, *p* = 0.9494).

#### 3.5.2. Correlational Analysis of Time-to-Peak-Variability (TTPV) Values and Ejection Fraction (EF) Among Gestational Age Groups

Although we noticed a weak positive monotonic correlation between the time to peak variability measured for the left ventricle and ejection fraction (ρ = 0.25) in group 1, this correlation was not statistically significant at the 0.05 level. In the other gestational age groups, the correlations between time-to-peak-variability (TTPV) values and ejection fraction (EF) were very weak and not statistically significant (Table 5).

## 4. Discussion

Current studies do not provide complete information regarding speckle tracking values throughout pregnancy for fetuses without a cardiac pathology. Moreover, some of the results related to the variation in the values for longitudinal strain with increasing gestational age are contradictory, as can be seen in the review study published by Oostrum et al. These shortcomings are due to the fact that in many studies, the number of included fetuses was low, and the technical conditions for acquiring the images were not optimal [10]. The use of speckle tracking is temporal resolution-dependent. During a cardiac cycle, the pattern in the ultrasound signal is tracked frame by frame. In children and adults, the recommended frame rate considered adequate for assessing myocardial deformation is approximately 40–80 frame rates (fps) [11]. In the small fetal heart, the frame rate is not well defined. Nevertheless, due to the increased fetal heart rate, the European Association of Cardiovascular Imaging and the American Society of Echocardiography suggest using the highest possible frame rate, preferably above 90 fps. Not using enough frames per fetal cardiac cycle can lead to errors in estimating myocardial strain [12]. In the current study, we performed an analysis of the longitudinal strain parameters for both ventricles on a significant number of healthy fetuses with optimal growth, which we divided into three gestational age groups. The analyzed images were recorded in optimal technical conditions, with the average value of the frame rate used being over 85 fps for each age group. The average value of the frame rate was inversely proportional to the gestational age, so, at a high gestational age, the quality of the analyzed images was lower.

In this study, we analyzed the LV function by means of classical echocardiography, more specifically, the calculation of the EF by the volumetric method, as well as by speckle tracing. In the specialized literature, there are only a few published studies regarding the evaluation of the systolic function of the LV by calculating the EF during the fetal period. In this study, as in the one published by Hamill et al., the volume of the left and the right ventricle, both in systole and diastole, increases with gestational age [13]. On the other hand, according to the aforementioned study, the EF of the LV decreases with increasing gestational age [13]. In the present study, the EF values obtained fall within the range of 52.2–64.4%. These values are close to those considered normal for newborns and infants 60 ± 2.7 [14]. Unlike the study published by Hamill, in this study, we did not observe variations in this parameter with gestational age. Although EF has lower values at a high gestational age, this difference has no statistical significance. The methods of determining the ventricular volumes are different in the two studies. Major differences were found between the technique of obtaining 4-chamber images and the calculation of the EF: in the study published by Hamill, a less standardized method was used [13]. Schubert et al. determined the LV ejection fraction in 30 fetuses, using the Simpson method, with the value being close to the one obtained in the present study [15].

The evaluation of the longitudinal deformation was carried out with the pGLS parameter for the entire LV, as well as per segment according to the methods mentioned above in the Materials and Methods section. The reference values can be found in Table 3. In comparison with studies published in the literature, although the interval obtained in our study is narrower, it fits into interval values published in other studies [16,17,18,19]. Regarding the EF, we did not obtain any statistically significant variations with gestational age. Although most of the published studies emphasize global values and focus less on segmental values, we consider it important to obtain reference values for segmental deformation. Certain cardiac pathologies can have an impact on the segmental parameters, especially at the level of the interventricular septum, without significant changes being seen with regard to global parameters.

For RV longitudinal strain, we obtained an interval for p RVGLS of –18.99 to −6.35 (Table 3). This interval falls in the range of the values concluded in the study published by Oostrum et al., with the interval determined in the present study being narrower [10].

In the intrauterine period, the RV is the dominant ventricle, having a larger pre- and afterload compared to the LV. The longitudinal strain does not capture this aspect, taking into account the fact that due to the immaturity of the fetal and neonatal myocardium, an increase in preload does not result in an increase in contractility. The stability of RV strain values was also observed in longitudinal studies, such as the one performed by Maskatia et al., who followed 60 pregnant women between 20 and 38 weeks of gestation [16], and also several cross-sectional studies [19,20,21].

Likewise, there are studies that reveal a decrease in longitudinal strain in the last trimester of pregnancy, such as the one conducted by Alsolai et al., who followed 276 pregnant women from the 36th week of pregnancy until the moment of birth [22]. This can be explained by the increase in RV afterload with a physiological restriction of PFO and PDA. This aspect was not taken into account in this study, considering that the maximum gestational age of fetuses included in this study was 39 weeks.

We also took into account the variability of the times in which the ventricular segments reached the maximum stretch, expressed by the LVTTPS and RVTTPS parameters. Several studies in the literature demonstrate that the high variability between the elongation peak of the segments affects the ventricular function [23]. In the present study, in which healthy fetuses were analyzed, we did not obtain a statistically significant negative correlation between EF and LVTTPS and RVTTPS. In this study, we also evaluated the reproducibility between two observers (Table 4). The degree of agreement in measuring the longitudinal strain between the two observers was very good.

### Limitations

Although prospective by design (with patients recruited and included during the data collection phase), with the inclusion of a significant but moderate number of fetuses, the main shortcoming of this study is the fact that it is not a longitudinal study. The reference intervals were derived from a single-center cohort, which may limit the generalizability to broader populations.

## 5. Conclusions

The reference values for longitudinal strain, expressed by the parameter pGLS for LV, were, for 22–27 gestational weeks, −20.79 to −8.05; for 28–33 gestational weeks, −20.14 to −8.99; and for 34–39 gestational weeks, −20.19 to −8.88. For pRVGLS, they were, for 22–27 gestational weeks, −18.99 to −6.35; for 28–33 gestational weeks, −18.93 to −6.35; and for 34–39 gestational weeks, −16.98 to −7.80. Up to the gestational age of 39 weeks, there were no statistically significant differences between gestational age groups.

## Figures and Tables

**Table 1:** Anthropometric and echocardiographic characteristics stratified by gestational age group.

	Gestational Age Group			
	Group 1 (n_1_ = 46)	Group 2 (n_2_ = 55)	Group 3 (n_3_ = 49)	*p* ^(a)^
Maternal age (years), mean (SD)	29.20 (4.62)	28.53 (2.92)	27.57 (3.58)	0.1389
BPD (%), mean (SD)	64.37 (12.92)	63.36 (11.59)	62.81(11.85)	0.8170
FL (%), mean (SD)	39.97 (10.13)	39.68 (10.96)	38.12 (9.15)	0.6270
Frame rate, median [IQR]	104 [100, 119.5]	98 [90, 140]	88 [81, 90]	<0.0001 *
Ejection fraction (EF, %)	59.38 (9.51)	57.10 (8.69)	55.65 (9.42)	0.1420

Group 1, 22–27 weeks; Group 2, 28–33 weeks; Group 3, 34–39 weeks; n, number of cases; SD, sample standard deviation; IQR, interquartile interval; FL, femur length; BPD, biparietal diameter; EF, ejection fraction; ^(a)^ estimated by Welch-F test or one-way ANOVA test or Kruskal–Wallis test; * a *p*-value < 0.05 was considered statistically significant.

**Table 2:** Speckle tracking-based regional strain measurements stratified by gestational age groups.

Measurements		Centrality and Dispersion Measures				Adjusted-*p* ^(b)^
	Gestational Age Groups	Mean (SD)	[Min, Max] Range	Median [IQR]	Estimated Marginal Means ^(a)^ (ES)	
LV Basal (%)	Group 1 (n_1_ = 46)	−17.1 (5.6)	[−37.4, −8.1]	−15.5 [−20.4, −13.4]	−17.1 (0.7)	0.9719 ^(1)^
	Group 2 (n_2_ = 55)	−15.4 (3.4)	[−24.3, −8.7]	−15.0 [−17.3, −13.0]	−15.4 (0.6)	0.9973 ^(2)^
	Group 3 (n_3_ = 49)	−15.7 (4.2)	[−26.5, −3.1]	−15.7 [−18.5, −12.3]	−15.6 (0.6)	1.0000 ^(3)^
LV Medial (%)	Group 1 (n_1_ = 46)	−15.7 (5.2)	[−36.7, −7.9]	−14.3 [−18.2, −12.5]	−15.7 (0.6)	1.0000 ^(1)^
	Group 2 (n_2_ = 55)	−14.8 (3.2)	[−21.8, −9.3]	−14.2 [−16.8, −12.9]	−14.9 (0.6)	1.0000 ^(2)^
	Group 3 (n_3_ = 49)	−15.5 (3.8)	[−23.3, −8.9]	−14.6 [−17.1, −13.5]	−15.5 (0.6)	1.0000 ^(3)^
LV Apical (%)	Group 1 (n_1_ = 46)	−16.8 (5.0)	[−33.0, −9.1]	−15.7 [−19.9, −13.3]	−16.8 (0.7)	1.0000 ^(1)^
	Group 2 (n_2_ = 55)	−16.1 (4.0)	[−25.6, −9.8]	−16.0 [−19.2, −12.3]	−16.2 (0.6)	0.9411 ^(2)^
	Group 3 (n_3_ = 49)	−15.0 (4.4)	[−26.1, −5.8]	−14.8 [−17.1, −11.7]	−14.9 (0.6)	0.9998 ^(3)^
Inter V Basal (%)	Group 1 (n_1_ = 46)	−13.8 (3.0)	[−20.7, −9.8]	−13.4 [−15.6, −11.6]	−15.5 (0.6)	0.9932 ^(1)^
	Group 2 (n_2_ = 55)	−15.6 (4.8)	[−36.2, −7.0]	−14.0 [−17.6, −12.8]	−14.1 (0.6)	1.0000 ^(2)^
	Group 3 (n_3_ = 49)	−14.8 (3.9)	[−27.6, −8.3]	−14.2 [−16.6, −12.6]	−14.7 (0.6)	1.0000 ^(3)^
Inter V Medial (%)	Group 1 (n_1_ = 46)	−15.4 (5.2)	[−36.3, −9.7]	−14.4 [−16.4, −12.2]	−15.4 (0.6)	1.0000 ^(1)^
	Group 2 (n_2_ = 55)	−14.7 (3.1)	[−23.5, −10.0]	−14.1 [−16.0, −12.4]	−14.7 (0.5)	1.0000 ^(2)^
	Group 3 (n_3_ = 49)	−15.2 (3.2)	[−23.3, −8.8]	−14.1 [−16.0, −12.4]	−15.1 (0.5)	1.0000 ^(3)^
Inter V Apical (%)	Group 1 (n_1_ = 46)	−14.8 (5.2)	[−36.2, −3.8]	−13.9 [−16.9, −12.0]	−15.8 (0.7)	1.0000 ^(1)^
	Group 2 (n_2_ = 55)	−15.2 (4.9)	[−26.8, −7.2]	−14.0 [−18.0, −11.2]	−14.7 (0.7)	0.9998 ^(2)^
	Group 3 (n_3_ = 49)	−14.9 (5.2)	[−24.9, −5.6]	−14.1 [−18.0, −11.2]	−14.4 (0.7)	1.0000 ^(3)^
RV Basal (%)	Group 1 (n_1_ = 46)	−15.8 (5.3)	[−34.4, −5.0]	−15.8 [−18.0, −11.9]	−15.8 (0.6)	1.0000 ^(1)^
	Group 2 (n_2_ = 55)	−15.4 (4.3)	[−28.2, −8.3]	−15.5 [−17.7, −12.4]	−15.5 (0.6)	0.9999 ^(2)^
	Group 3 (n_3_ = 49)	−14.6 (3.4)	[−27.3, −9.9]	−14.4 [−16.2, −12.2]	−14.6 (0.6)	1.0000 ^(3)^
RV Medial (%)	Group 1 (n_1_ = 46)	−13.0 (4.9)	[−32.1, −6.7]	−12.8 [−14.8, −9.4]	−13.0 (0.6)	1.0000 ^(1)^
	Group 2 (n_2_ = 55)	−12.5 (4.0)	[−25.6, −6.3]	−12.2 [−15.2, −9.4]	−12.5 (0.5)	1.0000 ^(2)^
	Group 3 (n_3_ = 49)	−12.7 (2.7)	[−21.7, −8.1]	−12.6 [−14.7, −11.2]	−12.7 (0.6)	1.0000 ^(3)^
RV Apical (%)	Group 1 (n_1_ = 46)	−12.8 (5.4)	[−31.6, −2.1]	−12.0 [−14.2, −9.7]	−13.0 (0.6)	1.0000 ^(1)^
	Group 2 (n_2_ = 55)	−11.9 (3.9)	[−22.7, −6.0]	−11.1 [−13.8, −9.1]	−12.5 (0.5)	1.0000 ^(2)^
	Group 3 (n_3_ = 49)	−11.6 (3.1)	[−19.3, −5.1]	−11.7 [−13.3, −9.5]	−12.7 (0.6)	1.0000 ^(3)^
RV pGLS (%)	Group 1 (n_1_ = 46)	−13.4 (4.5)	[−32.3, −7.0]	−15.2 [−15.1, −10.0]	−13.4 (0.5)	0.8222 ^(1)^
	Group 2 (n_2_ = 55)	−12.9 (3.6)	[−23.9, −7.4]	−12.7 [−15.4, −9.9]	−13.0 (0.5)	0.5658 ^(2)^
	Group 3 (n_3_ = 49)	−12.7 (2.6)	[−23.1, −8.2]	−12.2 [−14.3, −10.7]	−12.7 (0.5)	0.8906 ^(3)^
LV pGLS (%)	Group 1 (n_1_ = 46)	−15.8 (4.8)	[−36.0, −10.6]	−14.2 [−18.3, −12.5]	−15.8 (0.5)	0.2709 ^(1)^
	Group 2 (n_2_ = 55)	−14.7 (2.7)	[−20.1, −10.6]	−14.5 [−16.0, −12.4]	−14.7 (0.5)	0.5576 ^(2)^
	Group 3 (n_3_ = 49)	−15.1 (3.2)	[−23.2, −9.7]	−14.7 [−16.3, −13.2]	−15.1 (0.5)	0.8727 ^(3)^
EDV (mL)	Group 1 (n_1_ = 46)	0.9 (0.4)	[0.4, 2.0]	0.7 [0.5, 1.0]	0.9 (0.1)	<0.0001 * ^(1)^
	Group 2 (n_2_ = 55)	1.6 (0.6)	[0.4, 3.1]	1.4 [1.2, 2.1]	1.6 (0.1)	<0.0001 * ^(2)^
	Group 3 (n_3_ = 49)	2.3 (0.9)	[1.0, 5.7]	2.1 [1.6, 2.9]	2.3 (0.1)	<0.0001 * ^(3)^
ESV (mL)	Group 1 (n_1_ = 46)	0.3 (0.2)	[0.1, 0.9]	0.3 [0.2, 0.4]	0.3 (0.1)	<0.0001 * ^(1)^
	Group 2 (n_2_ = 55)	0.7 (0.4)	[−0.7, 1.7]	0.6 [0.4, 1.7]	0.7 (0.1)	<0.0001 * ^(2)^
	Group 3 (n_3_ = 49)	1.0 (0.6)	[0.4, 3.4]	0.9 [0.8, 1.2]	1.0 (0.1)	<0.0001 * ^(3)^

Group 1, 22–27 weeks; Group 2, 28–33 weeks; Group 3, 34–39 weeks; SD, sample standard deviation; LV, left ventricle; Inter V, interventricular septum; RV, right ventricle; RV pGLS, right-ventricle peak global longitudinal strain; EDV, end-diastolic volume; ESV, end-systolic volume; ^(a)^ estimated based on test models—mixed-design ANOVA or Welch-F test or one-way ANOVA test; ^(b)^ estimated from post hoc pairwise tests based on the Tukey method; ^(1)^ comparison between group 1 and group 2; ^(2)^ comparison between group 1 and group 3; ^(3)^ comparison between group 2 and group 3; * significant result with *p* < 0.05.

**Table 3:** Interobserver agreement analysis for echocardiographic variables.

Echocardiographic Characteristics	Observer 1	Observer 2	Interobserver Reproducibility (n = 20)	
	Mean (SD)	Mean (SD)	ICC [95% CI]	Systematic Bias [95% CI] Bland–Altman
LV Basal (%)	−16.39 (5.10)	−17.55 (4.02)	0.81 [0.57, 0.92]	1.16 [−0.10, 2.41]
LV Medial (%)	−15.91(4.30)	−16.28 (3.08)	0.76 [0.50, 0.90]	0.37 [−0.85, 1.59]
LV Apical (%)	−16.54 (5.03)	−16.96 (4.33)	0.80 [0.55, 0.91]	0.43 [−0.99, 1.85]
Inter V Basal (%)	−15.04 (4.79)	−15.69 (3.79)	0.86 [0.69, 0.94]	0.66 [−0.38, 1.69]
Inter V Medial (%)	−15.02 (4.45)	−15.41 (3.63)	0.75 [0.46, 0.89]	0.40 [−0.98, 1.77]
Inter V Apical (%)	−15.41 (5.86)	−16.11 (5.26)	0.81 [0.59, 0.92]	0.71 [−0.91, 2.32]
RV Basal (%)	−15.81 (3.63)	−15.09 (3.16)	0.64 [0.30, 0.84]	−0.73 [−2.07, 0.62]
RV Medial (%)	−12.43 (3.70)	−12.38 (3.24)	0.69 [0.36, 0.87]	−0.06 [−1.35, 1.24]
RV Apical (%)	−12.11 (4.17)	−11.03 (3.45)	0.77 [0.49, 0.90]	−1.08 [−2.24, 0.09]
RV pGLS (%)	−13.10 (3.23)	−12.74 (3.11)	0.85 [0.65, 0.94]	−0.36 [−1.42, 0.70]
EDV (mL)	1.62 (0.75)	1.54 (0.72)	0.78 [0.52, 0.91]	0.08 [−0.15, 0.31]
ESV (mL)	0.72 (0.47)	0.64 (0.33)	0.65 [0.30, 0.84]	0.08 [−0.08, 0.23]
EF (%)	56.87 (9.52)	57.95 (9.83)	0.75 [0.47, 0.89]	−1.09 [−4.32, 2.15]

LV, left ventricle; Inter V, interventricular septum; RV, right ventricle; RV pGLS, right-ventricle peak global longitudinal strain; EDV, end-diastolic volume; ESV, end-systolic volume; EF, ejection fraction; SD, sample standard deviation; ICC, Intraclass Correlation Coefficient type 2; bias, mean of the differences; 95% CI, 95% confidence interval.

**Table 4:** References ranges of strain measurements according to gestational age group.

	Gestational Age Groups					
	22–27 Weeks (n_1_ = 46)		28–33 Weeks (n_2_ = 55)		34–39 Weeks (n_3_ = 49)	
	95% Reference Interval (Estimated by Robust Method)					
Strain Measurements	Lower Limit [90% CI]	Upper Limit [90% CI]	Lower Limit [90% CI]	Upper Limit [90% CI]	Lower Limit [90% CI]	Upper Limit [90% CI]
LV Basal ^(a)^	−23.35 [−24.90, −21.17]	−8.04 [−9.33, −6.61]	−21.19 [−22.29, −19.98]	−8.84 [−9.97, −7.73]	−22.96 [−24.31, −21.53]	−8.48 [−9.82, −7.30]
LV Medial ^(b)^	−21.32 [−23.20, −19.54]	−6.90 [−8.49, −5.92]	−21.18 [−22.49, −19.56]	−8.00 [−9.16, −7.01]	−22.42 [−24.15, −20.49]	−7.39 [−8.63, −6.22]
LV Apical ^(c)^	−24.45 [−27.30, −23.05]	−6.83 [−8.72, −5.11]	−25.62 [−25.61, −22.53]	−7.76 [−9.07, −6.33]	−22.90 [−24.65, −21.06]	−6.61 [−8.15, −5.03]
Inter V Basal ^(d)^	−21.21 [−22.73, −18.99]	−7.45 [−8.61, −6.19]	−20.17 [−21.48, −18.74]	−7.77 [−8.81, −6.89]	−20.06 [−21.18, −18.82]	−8.03 [−9.27, −6.96]
Inter V Medial ^(e)^	−20.79 [−22.60, −19.06]	−7.05 [−8.31, −5.88]	−19.68 [−20.91, −18.29]	−8.46 [−9.44, −7.68]	−19.64 [−20.70, −18.51]	−9.70 [−10.66, −8.81]
Inter V Apical ^(f)^	−24.31 [−26.42, −22.28]	−5.60 [−7.41, −3.99]	−23.07 [−25.40, −20.65]	−3.37 [−5.33, −1.78]	−21.71 [−23.24, −20.17]	−6.32 [−7.87, −4.95]
RV Basal ^(g)^	−22.70 [−24.20, −21.17]	−7.36 [−9.14, −5.95]	−22.64 [−24.00, −21.20]	−7.24 [−8.72, −5.93]	−19.99 [−21.15, −18.72]	−8.43 [−9.48, −7.35]
RV Medial ^(h)^	−19.32 [−20.95, −17.65]	−4.75 [−6.12, −3.40]	−19.41 [−20.71, −17.97]	−4.96 [−6.17, −3.76]	−17.37 [−18.21, −16.42]	−7.65 [−8.65, −6.78]
RV Apical ^(i)^	−19.43 [−21.51, −17.41]	−3.80 [−5.26, −2.20]	−17.17 [−18.45, −15.65]	−4.77 [−5.83, −3.65]	−17.72 [−18.68, −16.51]	−5.61 [−6.91, −4.27]
RV pGLS ^(j)^	−18.99 [−20.31, −17.71]	−6.35 [−7.58, −5.13]	−18.93 [−20.39, −17.93]	−6.35 [−7.36, −5.22]	−16.98 [−17.83, −15.95]	−7.80 [−8.67, −6.99]
pGLS ^(k)^	−20.79 [−22.40, −18.88]	−8.05 [−9.26, −7.15]	−20.14 [−21.22, −18.88]	−8.99 [−9.77, −8.05]	−20.19 [−21.42, −18.93]	−8.88 [−10.03, −7.91]
EDV ^(l)^	0.13 [0.01, 0.27]	1.35 [1.17, 1.49]	0.34 [0.14, 0.56]	2.63 [2.33, 2.86]	0.48 [0.15, 0.75]	3.81 [3.36, 4.16]
ESV ^(m)^	0.04 [−0.05, 0.08]	0.53 [0.47, 0.60]	0.11 [0.003, 0.20]	1.12 [1.01, 1.23]	0.03 [−0.10, 0.22]	1.69 [1.53, 1.93]
EF ^(n)^	45.47 [42.28, 48.85]	75.57 [72.25, 78.59]	40.52 [37.42, 44.38]	74.69 [71.76, 77.51]	36.61 [32.87, 40.91]	75.33 [71.92, 78.93]

LV, left ventricle; Inter V, interventricular septum; RV, right ventricle; RV pGLS, right-ventricle peak global longitudinal strain; EDV, end-diastolic volume; ESV, end-systolic volume; EF, ejection fraction. The units of all strain measurements were percentages (%);the 95% reference interval was calculated by the robust method with 90% confidence limits estimated by the bootstrap percentile method (R = 5000) by removing outliers determined by Mark van der Loo’s method; after outlier removal: ^(a)^ n_1_ = 42; n_2_ = 53, n_3_ = 47; ^(b)^ n_1_ = 43; n_2_ = 55; n_3_ = 48; ^(c)^ n_1_ = 45; n_2_ = 55; n_3_ = 47; ^(d)^ n_1_ = 43; n_2_ = 54; n_3_ = 46; ^(e)^ n_1_ = 43; n_2_ = 53; n_3_ = 45; ^(f)^ n_1_ = 45; n_2_ = 52; n_3_ = 47; ^(g)^ n_1_ = 42; n_2_ = 53; n_3_ = 48; ^(h)^ n_1_ = 44; n_2_ = 54; n_3_ = 48; ^(i)^ n_1_ = 43; n_2_ = 51; n_3_ = 48; ^(j)^ n_1_ = 44; n_2_ = 53; n_3_ = 48; ^(k)^ n_1_ = 43; n_2_ = 55; n_3_ = 47; ^(l)^ n_1_ = 43; n_2_ = 53; n_3_ = 48; ^(m)^ n_1_ = 42; n_2_ = 51; n_3_ = 47; ^(n)^ n_1_ = 42; n_2_ = 54; n_3_ = 49.

**Table 5:** Correlations coefficients between ejection fraction and time to peak variability measured for the left and right ventricle.

	LV TTPV and EF		RV TTPV and EF	
Gestational Age Groups	Rho (ρ)	*p*	Rho (ρ)	*p*
Group 1	0.25	0.1013	0.11	0.4544
Group 2	0.14	0.3205	0.12	0.3710
Group 3	0.01	0.9516	−0.03	0.8386

Group 1, 22–27 weeks; Group 2, 28–33 weeks; Group 3, 34–39 weeks; LV, left ventricle; RV, right ventricle; TTPV, time to peak variability; EF, ejection fraction; ρ, Spearman correlation coefficient.

## Data Availability

The raw data presented in this study can be obtained upon reasonable request addressed to Cerghit-Paler Andreea at palerandreea@yahoo.com.
